# Effects of Dietary Supplementation of *Salvia officinalis* L. in Organic Laying Hens on Egg Quality, Yolk Oxidative Stability and Eggshell Microbiological Counts

**DOI:** 10.3390/ani11092502

**Published:** 2021-08-26

**Authors:** Dimitrios Galamatis, Georgios A. Papadopoulos, Diamanto Lazari, Dimitrios Fletouris, Evanthia Petridou, Georgios I. Arsenos, Paschalis Fortomaris

**Affiliations:** 1 Laboratory of Animal Husbandry, Faculty of Veterinary Medicine, School of Health Sciences, Aristotle University of Thessaloniki, 54124 Thessaloniki, Greece; geopaps@vet.auth.gr (G.A.P.); arsenosg@vet.auth.gr (G.I.A.); fortomap@vet.auth.gr (P.F.); 2 Hellenic Agricultural Organization DIMITRA (ELGO DIMITRA), General Management Assurance of Agricultural Products Quality, Kifissias 33 Str., 54248 Thessaloniki, Greece; 3 Laboratory of Pharmacognosy, Faculty of Pharmacy, School of Health Sciences, Aristotle University of Thessaloniki, 54124 Thessaloniki, Greece; dlazari@pharm.auth.gr; 4 Laboratory of Milk Hygiene and Technology, Faculty of Veterinary Medicine, School of Health Sciences, Aristotle University of Thessaloniki, 54124 Thessaloniki, Greece; djflet@vet.auth.gr; 5 Laboratory of Microbiology, Faculty of Veterinary Medicine, School of Health Sciences, Aristotle University of Thessaloniki, 54124 Thessaloniki, Greece; epetri@vet.auth.gr

**Keywords:** organic, aromatic plants, laying hens, *Salvia officinalis* L., malondialdehyde (MDA), *Enterobacteriaceae*

## Abstract

**Simple Summary:**

Organic laying hen systems are considered welfare-friendly, because hens are raised mainly outdoors under natural conditions. The notion is that eggs produced in those systems are better in terms of quality. Research has found that aromatic plants and their extracts can tackle many of the latter challenges when added to poultry diets due to their antioxidant and antimicrobial effects. The current study investigated the effects of a dietary supplementation of *Salvia officinalis* L. in organically raised laying hens during two experimental periods. The results showed improved oxidative stability of the eggs and reduced microbial counts in the eggshells. The egg quality parameters were partly affected, with the yolk weight showing the largest differences between treatments.

**Abstract:**

Aromatic plants of Labiatae family are used in poultry diets because of their antimicrobial and antioxidant activity. The notion is that hens raised in organic systems face several health and environmental challenges. Hence, the objective here was to assess hens’ performances and the quality of their eggs in such systems following a dietary supplementation of *Salvia officinalis* L. in powder form. The experiments were conducted over two successive years (1 and 2). They lasted 16 weeks each and involved 198 laying hens aged 40 weeks old randomly assigned to three groups: Con (control diet), Sal-0.5%, and Sal-1.0% (diets supplemented with *Salvia officinalis* L. at 0.5% or 1.0%, respectively). The malondialdehyde (MDA) levels in egg yolks in year 2 were lower in both Sal-0.5% and Sal-1.0% compared to the Con (*p* < 0.05). The total number of *Enterobacteriaceae* in eggshells were lower in Sal-1.0% compared to the Con (*p* < 0.05) in both years. The results suggest that a dietary supplementation of *Salvia officinalis* L. at 1.0% improves the antioxidant status and reduces the microbial load of eggs produced in organic systems.

## 1. Introduction

Egg production systems have evolved because of continuous efforts to ensure the health, balanced nutrition, and wellbeing of birds. Commercial egg production systems comprise confined systems, free range, and organic. The latter are considered as the ones providing the highest welfare status by both egg consumers and the public. Consumers’ knowledge and perception of egg production systems prioritizes animal welfare and dictates their purchasing decisions [1]. The prevailing view is that laying hens in free-range systems enjoy improved welfare compared to those housed indoors [2]. Free-range systems provide birds’ natural conditions to express their behaviors [3], and their eggs are considered as better in terms of quality [4]. However, it has been documented that a major challenge in these systems is the management of health-related issues [5], while in organic eggs, production welfare issues are under debate [6]. Furthermore, the evaluation of microbial contamination is also used to assess the egg quality [7]. Eggshells derived from alternative housing systems have been identified with a higher level of microorganisms than the ones collected from cage systems [7]. Thus, there is a necessity to produce eggs from organic systems with limited microbial contamination.

The ban of antibiotics as growth promoters (AGP) in animal feeds by the EU [8] forced research to seek alternative methods to cater the need of improving animal performances in livestock systems. The latter also became a priority in organic poultry production, seeking alternatives to control diseases and improve bird performances [9]. Hence, phytogenic feed additives, organic acids, and probiotics became the favorable nonantibiotic growth promoters considering their established use in animal nutrition [10]. Probiotic supplementation in organic laying hens’ diets influenced positively in the gut of hens the counts of beneficial bacteria such as *Lactobacillus* spp. and *Bifidobacterium* spp. and reduced the counts of harmful bacteria such as *E. coli*, clostridia, and staphylococci [11]. In the latter study, however, the level of eggshell contamination was not evaluated.

Essential oils and botanicals could serve the same scope as feed ingredients [12]. There is abundant evidence in the literature that aromatic plants and their extracts have antioxidant [9,13] and antimicrobial effects [14,15,16] following their supplementation in poultry diets. Moreover, such dietary supplements have a positive impact on the gastrointestinal function and nutrient digestibility [17]. Aromatic plants of the Labiatae family have been used in poultry diets due to their antimicrobial and antioxidant activity [9]. According to Bozkurt et al. [18], *Salvia officinalis* L., thyme, and oregano are among the most promising plants of the Labiatae family. The supplementation of *Salvia officinalis* L. extracts in roosters’ diets had positive effects on the quantity and quality of the sperm produced, while it increased the testosterone levels [19]. The dietary supplementation of such an extract reduced the *Salmonella* counts in the liver, spleen, and cecum of broilers infected with *Salmonella enteritidis* [20]. Levkut et al. [21] and Farhadi et al. [22] showed that the incorporation of *Salvia officinalis* L. extract in the broilers’ diet significantly improved their average daily gain and other performance parameters, whereas, in another study, Ryzner et al. [23] showed that it reduced the oxidative stress parameters evaluated in the red blood cells and kidneys of broilers. Recently, the supplementation of *Salvia officinalis* L. aqueous leaf extract in broilers’ diets enhanced the immunity response of broilers and significantly reduced the ileal counts of *E. coli* [24]. Elsewhere, the supplementation of *Salvia officinalis* essential oil in laying strain chicks’ diets showed that, when supplemented at lower concentrations, the antioxidant defense mechanisms were improved by the induction of antioxidant enzymes [25].

Considering the available literature, the evidence for the use of *Salvia officinalis* L. in the diets of organic laying hens is limited. Hence, the objective of the present study was to assess the egg quality parameters, oxidative stability of egg yolks, and microbial counts of eggshells of organically raised laying hens following the dietary supplementation of *Salvia officinalis* L. in powder form.

## 2. Materials and Methods

### 2.1. Animals and Housing

The experiment was conducted on a commercial organic laying hen farm located in Vasilika Village of Thessaloniki Prefecture, Central Macedonia, Greece. The farm was established in 1994 and started its commercial operation in 1996. Hy-Line Brown laying hens were housed under a free-range system in multiple buildings of the farm covering an area of 1500 m^2^. All hens had outdoor access to a field of 25,000 m^2^ planted with olive trees. The farm also had 200,000 m^2^ of cultivated land with cereals and other feeds following the organic agriculture guidelines used in the hens’ diet. All the feeds were formulated and produced in the mill of the farm. The farm organic registration code is 0EL54035. Organic certification was provided and monitored by an independent organization, “Physiologike” (https://physiologike.gr/; (accessed on 20 February 2021), according to EN ISO/IEC 17065.

### 2.2. Experimental Facility

**Figure 1:**
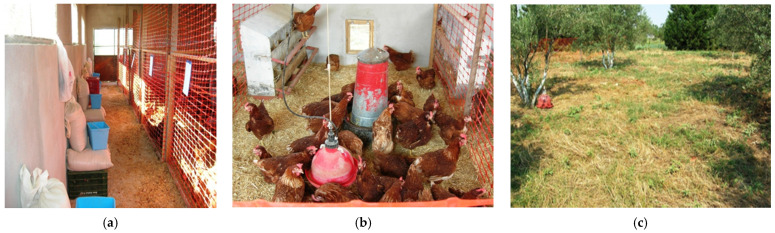
Indoor and outdoor parts of the experimental facility used in the study. (**a**) Corridor in front of the 6 experimental compartments. Experimental diets were kept in front of each respective compartment. (**b**) Inside view of an experimental compartment. Each compartment contained an egg nest with 8 available places, a conic feeder, and a bell-type drinker. A square pop hole enabled outdoor access of the laying hens. (**c**) Outside view of the field planted with olive trees in which laying hens had access. Bell-type drinkers were also placed in the field.

Laying hens were housed in a designated chamber with the dimensions 6 m × 12 m, covering a surface of 72 m^2^. The indoor facility was split into 6 compartments (dimensions 2 m × 4.2 m), each one covering a surface of 8.4 m^2^. The compartments were constructed and separated using a portable wooden structure together with a plastic net. Each compartment had an egg nest with 8 available places (1 place per 3 laying hens), a conic feeder with a capacity of 25 kg, and a bell type drinker with a diameter of 38 cm (Plasson Livestock, Maagan Michael, D.N. Menashe 3780500, Israel). To enable outdoor access for all laying hens, each compartment had a specially constructed square pop hole with the dimensions of 0.5 m × 0.5 m. Moreover, each compartment had a door to allow access to personnel for the everyday husbandry practices, e.g., egg collection and feed renewal (Figure 1). The designated chamber had 3 windows, with dimensions of 1.8 m length × 0.8 m height, to ensure proper air circulation. The chamber walls and all equipment used were pressure washed and disinfected before the start of each experiment. Sawdust was used for bedding. An 8-h darkness period was set, with the duration of physical light about 6 h, on average.

In each experiment, 198 Hy-Line brown laying hens approximately 40 weeks old were used. The laying hens were randomly assigned to 3 experimental groups (*n* = 66 per group; 2 pens of 33 laying hens per group) as follows: (i) group Con: hens were fed the standard organic diet and served as the control group, (ii) group Sal-0.5%: hens were fed the standard organic diet supplemented with dehydrated *Salvia officinalis* L. in powder form at the level of 0.5%, and (iii) group Sal-1.0%: hens were fed the standard organic diet supplemented with dehydrated *Salvia officinalis* L. in powder form at the level of 1.0%. The study was subdivided into two successive phases. The duration of each experimental phase was 16 weeks and started in July and ended in November of the same year. The weather data of the area where the farm is located were collected from the Hellenic National Meteorological Service (http://www.hnms.gr; accessed on 20 February 2021). The meteorological data analysis of the study period showed a higher average temperature and a lower average precipitation rate during the first experimental period (year 1).

### 2.3. Ethics Approval Statement and Experimental Design

The research protocol of the study was part of a PhD project of DG and was approved by the General Assembly of the Veterinary Faculty of Aristotle University of Thessaloniki. The General Assembly of the Veterinary Faculty of Aristotle University of Thessaloniki approved the specific PhD protocol in its decision: 55/27-5-2015.

### 2.4. Ingredient Sources, Diet Formulation, and Analysis

*Salvia officinalis* L., used in the present study, originated from plants that were cultivated in a field in Western Macedonia, Greece. The harvest was made each year at the beginning of June after blossom. The harvested plants were placed in designated buildings that allowed natural drying, a process that lasted approximately 15 days. Sprouts, leaves, and flowers were subsequently ground into a powder form and stored for future use in the experiment. Table 1 shows the main ingredients of the experimental diets and their nutrient analysis. The proximate analysis and chemical composition of the diets are provided in Table 2, whereas Table 3 shows the chemical composition (%) of the *Salvia officinalis* L. extract used in the experiment. The essential oil of *Salvia officinalis* L. was analyzed at the Laboratory of Pharmacognosy, School of Pharmacy, Faculty of Health Sciences, Aristotle University of Thessaloniki. The dried and powdered leaves of the plant, which were received by the company “DIOSKOURIDIS”, were subjected to water distillation for two hours in a Clevenger apparatus according to Europaea Pharmacopeia and connected to a modified refrigerated container of essential oils. Additional cooling was used in order to reduce the byproducts of the heat treatment. After distillation, the essential oil was taken up in 2 mL of pentane (GC grade) and filtered through anhydrous sodium sulfate to dehydrate it. The obtained essential oil was kept at −4 °C until it was analyzed. The essential oil yield was expressed in mL.100^−1^ g d.w. Essential oil analyses were performed on a Shimadzu GC-2010-GCMS-QP2010 system operating at 70 eV. This was equipped with a split/spitless injector (230 °C) and a fused silica HP-5 MS capillary column (30 m × 0.25 mm i.d., film thickness 0.25 μm). The temperature program was from 50–290 °C at a rate of 4 °C/min. Helium was used as a carrier gas at a flow rate of 1.0 mL/min. The injection volume of each sample was 1 μL. The retention indices (RI) for all the compounds were determined according to Van den Dool and Kratz [26], using n-alkanes as the standards. The identification of the components was based on a comparison of their mass spectra with those of NIST21 and NIST107 [27] and by comparison of their retention indices with the literature data [28]. The essential oils were often subjected to co-chromatography with authentic compounds (Fluka, Sigma).

The cultivated *Salvia officinalis* L. had a 2.37% extract concentration with the following basic components:

- *cis*- or α-Thujone (33.80%),
- *trans*- or β-Thujone (6.97%),
- 1,8-Cineole (=Eucalyptol) (11.61%), and
- Camphor (24.54%).

Approximately 98.95% of the composition of the extract was identified, and of 36 active substances, the concentration was analyzed (Table 3).

### 2.5. Laying Hen Performance

During both experimental periods, the feed intake of laying hens was measured on a weekly basis, and the average daily feed intake was calculated. The feed refusals and egg weight were recorded on a weekly basis to have an estimation of the average daily feed intake and egg mass production, respectively.

The daily egg production was also recorded for each experimental group by collecting eggs every morning (between 9:00–10:00 a.m.). The weekly egg-laying rate was expressed in % on a treatment basis. The feed conversion ratio was calculated by dividing the feed intake with the average egg weight for the respective periods and was expressed as kg of feed per kg of eggs produced. The individual body weight of hens was measured with a digital balance (Supra: SS3242, precision 5 g, Dinaksa Pesaje Industrial, Arrigorriaga—Vizcaya, Spain) at the start, the middle, and at the end of each experimental period.

### 2.6. Egg Quality

At weeks 2, 4, 6, 8, 10, 12, 14, and 16 of each experimental period, 12 eggs per treatment were collected to assess the egg quality parameters. The eggs were collected randomly from each treatment and weighed to calculate the average egg weight. The average egg mass was calculated on a weekly and on a pen basis by multiplying the egg weekly laying performance (%) with the average egg weight and dividing by 100: Egg mass = (Hen week % egg production * Egg Weight)/100. The eggs were weighed with a digital balance with 0.1-g accuracy (Navigator TM, N2B110, OHAUS Corporation, Parsippany, NJ, USA). The length and width of the eggs were measured with a digital caliper (EMC, LTD, China) with 0.01-mm accuracy. The egg shape index was calculated using the formula: shape index = (width/length) × 100. The eggshell color was measured with a reflectometer (EQ Reflectometer, York Electronics Centre, York, UK), while the egg-specific gravity was calculated using the method based on the Archimedes principle. The eggshell was washed to remove the adhering albumen and air-dried. The thickness of the eggshell with the membranes was measured with a caliper (accuracy 0.001 in, AMES, Waltham, MA, USA), while its weight was measured using a digital balance (Navigator TM, N2B110, OHAUS Corporation, Parsippany, NJ, USA). The albumen weight was calculated by subtracting the weights of the egg yolk and shell from the weight of the egg. The yolk color was estimated using the Roche Colour Yolk Fan, while the Haugh units were measured using designated equipment by the EQM York Electronics Center (Egg Quality Microprocessor, Technical Services & Supplies Ltd., Dunnington, York, UK). The egg quality parameters were measured at the Laboratory of Animal Husbandry, Faculty of Veterinary Medicine, Aristotle University of Thessaloniki.

### 2.7. Oxidative Parameters Analysis

#### 2.7.1. Iron-Induced Lipid Oxidation of Egg Yolk

The oxidative stability was assessed in 12 egg yolks from each treatment sampled at the end of each experimental period (36 eggs/experimental period in total) following iron-induced lipid oxidation. Twelve (12) yolks of each treatment were mixed to create four mixtures of three yolks each. Four 1-g samples from each of the four mixtures were weighed into 50-mL centrifuge tubes, and iron-induced lipid oxidation was carried out with a modification of the method of Galobart et al. [29]. According to that, 0.5 mL of 5-mM ferrous sulphate and 0.5 mL of 2-mM ascorbic acid were added to the yolk samples, and the contents of the tubes were vortex-mixed vigorously for 15 s. Following incubation at 37 °C for either 0, 50, 100, or 150 min, all yolk samples were immediately submitted to malondialdehyde (MDA) determination for evaluating the extent of lipid oxidation.

#### 2.7.2. Evaluation of Lipid Oxidation in Egg Yolk

The evaluation of lipid oxidation was based on malondialdehyde (MDA) determination using the selective third-order derivative spectrophotometric method developed by Botsoglou et al. [30]. According to this method, the yolk sample (12 egg yolks per treatment) was mixed with 5 mL of 0.8% butylated hydroxytoluene (Sigma Chemical, Co., St. Louis, MO, USA) in hexane and 8 mL of 5% aqueous trichloroacetic acid (Sigma Chemical, Co., St. Louis, MO, USA). The mixture was homogenized (Ultra-Turrax, Janke & Kunkel-IKA-Labortechnik, Staufen, Germany) for 30 s and centrifuged (Hettich Universal-1200) for 3 min at 2000× *g*. The top hexane layer was discarded, and a 2.5-mL aliquot of the bottom aqueous layer was mixed with 1.5 mL of 0.8% aqueous 2-thiobarbituric acid (Sigma Chemical, Co., St. Louis, MO, USA). The mixture was incubated at 70 °C for 30 min and, following cooling under tap water, was submitted to third-order derivative spectrophotometry (Shimadzu, Model UV-160A, Tokyo, Japan). The height of the peak at 521.5 nm was used for calculation of the MDA concentration in the yolk extracts based on the slope and intercept data of the computed least-squares fit of a freshly prepared standard calibration curve.

### 2.8. Microbiology Analysis

All samples were aseptically collected, placed in sterile bags, and transferred in cool bags to the Laboratory of Microbiology and Infectious Diseases, School of Veterinary Medicine, Aristotle University of Thessaloniki for bacteriological analyses. The detection of *Campylobacter* and *Salmonella* was performed according to ISO 10272-1 [31] and ISO 6579-1 [32], respectively. The detection and enumeration of *Enterobacteriaceae* was performed according to ISO 21528-2 [33]. For *Campylobacter* detection, three eggshells were separated from their contents and were aseptically placed and weighted in a sterile stomacher bag. A tenfold dilution was obtained by adding an enrichment medium Bolton broth. After homogenization, the samples were incubated in a microaerophilic atmosphere at 37 °C for 4 h and then at 41.5 °C for 24 h. From the enrichment culture, 10 μL were transferred and spread in the selective mCCD agar (OXOID) and incubated at 41.5 °C for 44 h in the above atmosphere. All plates were examined for the growth of suspected *Campylobacter* colonies. For *Salmonella* detection, 25 g of both eggshells and contents (yolk and albumen) of 2 eggs were weighted and pre-enriched in 225 mL of buffered peptone water (BPW; Biolife, Italy) at 37 °C for 18 h. Then, 100 μL were plated in 3 drops equally spaced onto the surface of Modified Semi-solid Rappaport Vassiliadis agar (MSRV, OXOID) and incubated at 41.5 °C for 24 h. Plates with no growth were additionally incubated for 24 h. All plates were examined for the growth of white grey colonies with a turbid zone around the droplet. Moreover, 1 mL of the pre-enriched culture was transferred to 10-mL Muller Kauffman tetrathionate/novobiocin broth (MKTTn-Biolife, Italian S.r.L, Milano, Italy) and incubated at 37 °C for 24 h. Suspected colonies from the MSRV plates, as well as a loop from MKTTn, was spread to XLD (Merck, Germany) and RAMBACH (Merck, Germany) agar plates. After the incubation at 37 °C for 24 h, the growth of typical or atypical *Salmonella* colonies was evaluated. For the detection and enumeration of *Enterobacteriaceae*, 10 g of the shell sample was transferred aseptically to 90 mL of diluent buffered peptone water (BPW). After homogenization, 1 mL of the initial dilution was transferred to 9 mL of diluent in the tubes. This resulted in 10^−2^ and 10^−3^ dilutions. Double-row Petri dishes (Ø 90 mm) were inoculated with a sterile pipette in 1 mL of the initial dilution, as well as the next 2 decimal dilutions. Approximately 10 mL of the Violet Red Bile Glucose agar (VRBG) substrate (Biolife, Italian S.r.L, Milan, Italy) was added to each plate. After complete solidification of the material, approximately 10 mL of the VRBG agar covering layer was added and incubated at 37 °C for 24 h ± 2 h. Pink to red or purple colonies were counted as *Enterobacteriaceae*, while 5 colonies from each plate was subjected to further biochemical identification according to the ISO methodology mentioned above.

### 2.9. Statistical Analysis

The data were analyzed using the Statistical Package for Social Sciences software (SPSS 25.0 Version, Chicago, IL, USA). Statistical significance was considered at *p* < 0.05, while a statistical trend was considered for those values between 0.05 < *p* < 1.0. The results were presented as the mean ± standard deviation (SD) where appropriate. Parameters were analyzed with one-way ANOVA, and post-hoc comparisons between treatments were made by Tukey’s test. Treatment was included in the model as the fixed factor. In accordance with the recently published study in organic laying hens by van der Heide et al. [34], which consisted of a similar experimental layout as the current study, the laying hen performance data were not subjected to statistical analysis due to a limited number of repetitions (*n* = 2 per treatment), and thus, only descriptive data are presented for years 1 and 2. The combined laying hen performance data for years 1 and 2 were analyzed for differences between the treatments. The egg quality data, egg lipid oxidation data and eggshell microbiology data were analyzed statistically, considering the sample (egg) as the statistical unit. The egg quality data and egg lipid oxidation were also analyzed with one-way ANOVA. Data on the microbiology analysis of the eggshells and, specifically, of the enumeration of *Enterobacteriaceae* were analyzed with a nonparametric Mann–Whitney test.

## 3. Results

### 3.1. Laying Hen Performance

The performance parameters during the first experimental period (year 1) are summarized in Table 4. The laying% and egg mass were numerically higher in the Con and Sal-1.0% groups compared to Sal-0.5%. The average daily feed intake (ADFI) was numerically higher in Sal-1.0% than Sal-0.5% and intermediate in the Con group. The feed conversion ratio (FCR) was numerically lower in the Con and Sal-1.0% groups than Sal-0.5%. The body weight of laying hens did not differ either between treatments at the start or at the end of the experimental period.

The results of the performance parameters during the second experimental period (year 2) are summarized in Table 5. The laying%, ADFI, and egg mass were numerically higher in the Sal-0.5% compared to the other two groups. The body weights of laying hens did not differ between treatments either at the start or at the end of the experimental period.

When investigating the results for both the experimental periods, it was revealed that the egg mass was higher in the Sal-0.5% than the Con group, while, in the Sal-1.0% group, it was intermediate (*p* = 0.008) (Table 6). The data on the FCR showed a better feed conversion in the Sal-0.5%compared to the Con group (*p* = 0.031).

### 3.2. Egg Quality Parameters

The treatment differences for the egg quality parameters were investigated separately for each year and for each sampling timepoint—namely, weeks 2, 4, 6, 8, 10, 12, 14, and 16. The results presented here to the overall average values obtained in each experimental period.

The results on the egg quality parameters during year 1 are summarized in Table 7. The egg weights tended to be greater in the Sal-1.0% group than the Con (*p* = 0.087). The yolk weight was higher in Sal-1.0% compared to Con, with the Sal-0.5% was intermediate (*p* = 0.043). The shell weight and egg width tended to greater in the Sal-1.0% than the other two groups (*p* = 0.060 and *p* = 0.067, respectively). The albumen and yolk pH were significantly higher in the Sal-1.0% compared to the Con (*p* = 0.001 and *p* = 0.002), while the yolk pH was also higher in the Sal-0.5% than the Con group.

The results on the egg quality parameters during year 2 are summarized in Table 8. The majority of the egg quality parameters evaluated were similar between the treatments, and significant differences appeared only for the albumen and yolk pH. Albumen pH was higher in the Sal-1.0% group compared to the other two groups and was higher in the Sal-0.5% than the Con group (*p* < 0.001). The yolk pH was higher in both groups supplemented with *Salvia officinalis* L. compared to the Con group (*p* < 0.001).

### 3.3. Oxidative Stability of Egg Yolk

The results of the extent of lipid oxidation (levels of malondialdehyde, MDA) for year 1 are presented in Table 9. No significant difference was detected between the treatments.

The results of the extent of lipid oxidation for year 2 are presented in Table 10. At 50 min and 100 min, the lipid oxidation was significantly lower in the Sal-1.0% group compared to the control one, while it was intermediate for the Sal-0.5% group (*p* = 0.034 and *p* = 0.038, respectively). At 150 min, the lipid oxidation was lower in both the groups supplemented with *Salvia officinalis* L. compared to the control one (*p* = 0.010).

### 3.4. Microbiological Analysis of Eggshells

The testing of both egg yolk and eggshell revealed the absence of *Salmonella* spp. and *Campylobacter* spp. during the first experiment. For the colonies of *Enterobacteriaceae*, their number per experimental group is presented in Table 11. The total number of *Enterobacteriaceae* was significantly lower in the Sal-1.0% group compared to the Con (*p* = 0.032).

Additionally, in year 2, the testing of both egg yolk and eggshell revealed the absence of *Salmonella* spp. and *Campylobacter* spp. during the second experiment. For the colonies of *Enterobacteriaceae*, their number per experimental group is presented in Table 12. The total number of Enterobacteriaceae was significantly lower in the Sal-1.0% group compared to the Con (*p* = 0.041) and tended to be lower in Sal-1.0% compared to Sal-0.5% (*p* = 0.087).

## 4. Discussion

The development in poultry production systems from traditional cages to enriched cages, free-range systems, and organic systems resulted in multiple challenges for the health and welfare of laying hens. The new systems also increased the efforts to maintain the high performance and health of hens [35]. As asserted in the Introduction, the supplementation of aromatic plants in the diet of laying hens has been a promising practice recently due to their antimicrobial–bacteriostatic and antioxidant properties [10]. However, in organic systems, there is limited evidence regarding the benefits of dietary supplementation of aromatic plants in production output and the health of laying hens [9]. Hence, in the present study, we chose to study the role of a common and distinctive aromatic plant, *Salvia officinalis* L., on the performance and health of laying hens raised in a commercial organic farm. The results showed that *Salvia officinalis* L. can improve certain egg quality characteristics subject to several environmental parameters at the farm level that are difficult to control in commercial farms [36]. Moreover, our intention was to avoid major alterations in the daily practices of the farm.

The primary focus of the study was on the egg quality, yolk oxidative stability, and eggshell microbiological counts. Although assessing the effects on the performance parameters was not within the main objectives, we presented performance data to provide more conclusive information for the readers. The practical aspects of the experimental setup of the study obliged us to split the 66 laying hens into two pens that were replicates of each treatment and for each year, resulting in 33 hens per pen and with two pens per treatment. Likewise, in a recent study with organic laying hens, each treatment comprised of two pens with 35 hens in each pen [34]. Similarly, in a study investigating the effects of probiotic supplementation in organic laying hens, each treatment comprised three replicates with 20 hens in each replicate [11]. Due to the housing requirements for organic laying hens for outdoor access, such an experimental setup for organic laying hens’ feed experiments is inevitable, as housing hens individually in experimental battery cages is not possible. Under these practical considerations, and in agreement with the study of van der Heide et al. [34], we decided not to proceed with the statistical evaluation of the performance data for years 1 and 2. Instead, we assessed the treatment differences for the performance data of both years. Still, this approach enabled the inclusion of four replicate performance data per treatment, which is not an optimum number of replicates for statistical comparisons. Nevertheless, the descriptive results in year 1 showed that the performances of the hens differed between the groups supplemented with *Salvia officinalis* L. at 0.5% compared to the controls, with the laying percentage lower and feed conversion ratio higher. This may be attributed to the increased feed intake in the specific group during the transition from the summer to the autumn period [35,37]. It is plausible that the external factors may have been more detrimental in this group compared to the other two groups. Mashaly et al. [38] and Bozkurt et al. [39] reported increased feed intakes in birds during periods of lower environmental temperatures following periods of higher temperatures. During the second experimental period (year 2), the performance parameters were similar between the experimental groups. However, the daily feed intake was higher for hens of all groups compared to the first year. This could be due to the higher levels of humidity noticed during year 2. Other factors could also be involved for the increased feed intake, especially in the groups supplemented with the aromatic plant, i.e., a positive influence on the feed palatability in the group supplemented with 0.5% of *Salvia officinalis* L. but not in that supplemented with 1% [13]. Bölükbası et al. [40] showed that the inclusion of a 200-mg/kg extract of *Salvia sclarea* L. reduced the feed consumption of laying hens. In the latter study, the feed conversion was also improved in the hens supplemented with *Salvia sclarea* L., but there was no effect on the body weight or laying performance. Moreover, the supplementation of *Salvia officinalis* L. leaves at 2.5% in the laying hens’ diet did not improve any of the performance parameters [41]. The supplementation of the extract of *Salvia sclarea* L. in the diets of laying hens did not affect the body weight or laying percentage but improved the feed conversion ratio [40]. The data on the overall experiment revealed that the hens supplemented with 0.5% of *Salvia officinalis* L. showed an improved feed conversion compared to the Con group, which corroborated with the previous findings. Meanwhile, a favorable increase in the egg mass for the overall experiment was noted in the Sal-0.5% compared to Con group, which can be attributed to the improvement of feed conversion in this specific *Salvia officinalis* L.-supplemented group. According to Çabuk et al. [42], the supplementation of a product containing extracts from various aromatic plants, including *Salvia triloba* L., in laying hens’ diets increased the weight of hens and improved the feed conversion ratio. Özek et al. [43] and Bozkurt et al. [39] used an extract also containing *Salvia triloba* L. in their experiment but did not find any effects on the performance of laying hens. It should be noted that the previous studies have been conducted in conventional systems and not in organic ones. Laying hens in organic production systems show greater feed conversion rates than those reared under conventional systems [44]. The latter difference is attributed to the greater level of activity and due to a greater variability of the environmental temperature in organic systems [44,45].

In our study, the yolk weight was increased in eggs from hens supplemented with 1.0% *Salvia officinalis* L. in both experiments compared to the other two groups. This effect could be attributed to the greater abundance of antioxidant substrates in a specific group, which helped hens to tolerate thermal stress during the production of the yolk [46,47]. The improved digestion and absorption of nutrients in the groups supplemented with *Salvia officinalis* L. could also have contributed to the higher yolk weight [48,49,50]. Similarly, Bozkurt et al. [39] reported that the yolk weight was improved when the diets of laying hens were supplemented with an extract containing, among others, *Salvia triloba* L. In the latter study, the hens were subjected to thermal stress conditions, and the outcome was an increase in yolk weight with a concurrent reduction in albumen weight. In the past, other have also investigated the effects of *Salvia officinalis* L. supplementation on the egg quality characteristics. Loetscher et al. [41] showed that the dietary supplementation of leaves of *Salvia officinalis* L. at 2.5% did not improve the egg quality parameters. On the other hand, the supplementation of an extract of *Salvia sclarea* L. increased the egg weight and Haugh units, while it reduced the yolk percentage [40]. Under thermal stress conditions, the supplementation of an extract containing *Salvia triloba* L. increased only the egg Haugh unit [42]. Elsewhere, aromatic plant supplementation of the family of Labiatae (oregano, thyme, and rosemary) increased the egg yolk weight [51].

In the present study, it is plausible that laying hens were subjected to chronic heat stress conditions. According to Akbarian et al. [52], chronic heat stress is a combination of high ambient temperatures during a prolonged period. This was probably the case in our study, with the average environmental temperatures being higher during the summer months and especially during the first experimental period. It is known that chronic heat stress induces a depletion of antioxidant reserves in poultry [52], and therefore, it is necessary to replace them by dietary means. Phytochemicals are among those dietary antioxidant ingredients beneficial in chronic heat-stressed poultry [52]. In our study, based on the results of MDA in the egg yolks during year 1, it can be hypothesized that chronic heat stress had a detrimental effect on the antioxidant mechanisms of laying hens supplemented with *Salvia officinalis* L. However, during year 2, the MDA levels in egg yolks were significantly lower in both groups supplemented with *Salvia officinalis* L. This finding suggests that chronic heat stress was less pronounced compared to year 1 and that, under these conditions, *Salvia officinalis* L. supplemented with either 0.5% or 1.0% was able to counteract the oxidative stress conditions. Loetscher et al. [41], showed that the supplementation of leaves of *Salvia officinalis* L. at a level of 2.5% improved the antioxidative properties of egg yolks. Elsewhere, it was shown that the supplementation of an extract also containing *Salvia triloba* L. resulted in a significant reduction of MDA in egg yolks and increased the levels of liver enzymes involved in the antioxidative pathways [53]. It is apparent that *Salvia officinalis* L. supplementation improves not only the antioxidative properties of the eggs but also protects laying hens from pro-oxidative conditions.

The results also showed that the dietary supplementation of *Salvia officinalis* L. at 1% significantly reduced the counts of Enterobacteriaceae in eggshells compared to the control group in both experimental periods. This effect could be attributed to the antibacterial properties of the *Salvia officinalis* L. components, such as α-thujone, which had a concentration of 40% in the plants used in our study. Previous studies showed that the supplementation of 200 mg/kg of extract of *Salvia sclarea* L. in laying hen diets reduced the Enterobacteriaceae counts in the feces [40]. These findings are important, as eggs collected from free-range systems were shown to be more contaminated with Enterobacteriaceae counts than those eggs collected from conventional ones [54,55,56].

## 5. Conclusions

The supplementation of *Salvia officinalis* L. in powder form, especially at a level of 1%, can improve the oxidative stability of eggs produced by laying hens raised in organic systems. The dietary treatments reduced significantly the counts of Enterobacteriaceae in the eggshells. The potential of the use of *Salvia officinalis* L. in a powder form in organic laying hens’ diets is promising and requires further investigation.

## Figures and Tables

**Table 1:** Gross ingredient composition (kg/1000 kg, as fed basis) of the experimental laying hen diets Con (control diet), Sal-0.5%, and Sal-1.0% (diets supplemented with *Salvia officinalis* L. at 0.5% or 1.0%, respectively).

Ingredients	Treatments		
	Con	Sal-0.5%	Sal-1.0%
Corn	518	510	505
Soybean-44%CP	238	240	242
Barley	100	98	93
Limestone	104.4	104.4	104.4
Monocalcium Phosphate	9.5	9.5	9.5
DL-Methionine	1.5	1.5	1.5
Choline	0.5	0.5	0.5
^1^ Premix	2	2	2
Soybean oil	22	25	28
*Salvia officinalis* L.	0	5	10
Sodium carbonate	2.3	2.3	2.3
Salt	1.8	1.8	1.8

^1^ per kg of diet: vitamin A, 10,000 I.U.; vitamin D3, 2500 I.U; vitamin E, 30 mg; vitamin K, 4 mg; vitamin B1, 1 mg; vitamin B2, 5 mg; vitamin Β6, 3 mg; vitamin B12, 0.02 mg; vitamin B3, 30 mg; vitamin B5, 10 mg; folic acid, 1 mg; biotin, 0.05 mg; vitamin C, 10 mg; choline, 400 mg; cobalt, 0.20 mg; copper, 10 mg; iodine, 1 mg; iron, 40 mg; manganese, 120 mg; selenium, 0.30 mg, zinc, 100 mg.

**Table 2:** Proximate analysis and chemical composition of the experimental diets of experimental laying hen diets: the Con (control diet), Sal-0.5%, and Sal-1.0% (diets supplemented with *Salvia officinalis* L. at 0.5% or 1.0%, respectively).

Nutrients	Treatments		
	Con	Sal-0.5%	Sal-1.0%
Crude protein	15.6	15.6	15.6
Crude fat	4.69	4.97	5.24
Crude fiber	3.08	3.07	3.06
Total calcium	4.17	4.17	4.17
Total phosphorus	0.51	0.51	0.51
Available phosphorus	0.32	0.32	0.32
Metabolizable Energy (Kcal/kg)	2808	2805	2803
Lysine	0.83	0.83	0.83
Methionine	0.41	0.41	0.41
Methionine-Cystine	0.68	0.68	0.68
Threonine	0.6	0.6	0.6
Tryptophan	0.18	0.18	0.18
Sodium	0.15	0.15	0.15
Chloride	0.14	0.14	0.14

**Table 3:** Chemical composition (%) of *Salvia officinalis* L. essential oil used in the experiment.

Chemical Compounds	RI ^a^	%	Method ^b^
Tricyclene	920	tr	AI, MS
α-Thujene	926	tr	AI, MS
α-Pinene	932	2.47	AI, MS, Co-GC
Camphene	946	2.46	AI, MS
β-Pinene	974	1.04	AI, MS, Co-GC
β-Myrcene	992	0.64	AI, MS, Co-GC
α-Phellandrene	1004	0.06	AI, MS
α-Terpinene	1016	0.11	AI, MS
p-Cymene	1024	0.51	AI, MS, Co-GC
Limonene	1028	1.54	AI, MS
1,8-Cineole (=Eucalyptol)	1030	11.61	AI, MS, Co-GC
γ-Terpinene	1059	0.17	AI, MS
*trans*-Linalool oxide (furanoid)	1073	tr	AI, MS
Terpinolene	1088	0.19	AI, MS
Linalool	1100	0.34	AI, MS, Co-GC
α-Thujone	1104	33.80	AI, MS
β-Thujone	1116	6.97	AI, MS
α-Campholenal	1126	tr	AI, MS
Isothujol	1135	0.09	AI, MS
*cis*-Sabinol	1140	0.07	AI, MS
Camphor	1143	24.54	AI, MS
Neoisothujol	1150	0.07	AI, MS
*trans*-Pinocamphone	1161	0.10	AI, MS
Borneol	1165	2.93	AI, MS, Co-GC
Menthol	1173	0.08	AI, MS
Terpinen-4-ol	1177	0.56	AI, MS, Co-GC
p-Cymen-8-ol	1186	0.08	AI, MS
α-Terpineol	1191	0.28	AI, MS
Myrtenol	1197	0.14	AI, MS
Isobornyl acetate	1286	1.73	AI, MS
*trans*-Pinocarvyl acetate	1294	0.23	AI, MS
β-Caryophyllene	1421	0.39	AI, MS, Co-GC
α-Caryophyllene	1455	1.34	AI, MS, Co-GC
Caryophyllene oxide	1586	0.20	AI, MS, Co-GC
Viridiflorol	1594	3.07	AI, MS
Humulene epoxide	1612	0.96	AI, MS

^a^ HP-5MS column. ^b^ Identification method: RΙ = Retention Index determined on a HP-5 MS capillary column using a homologous series of n-alkanes (C9-C25), MS = mass spectrum, Co-GC = co-injection with authentic compound, and tr = traces, concentrations <0.05.

**Table 4:** Effects of treatments on the performance parameters of laying hens during the 1st experimental period (*n* = 2 per treatment for the laying performance (%), ADFI, egg mass, and FCR; *n* = 66 per treatment for the body weight measurements).

Parameter	Treatments			
	Con	Sal-0.5%	Sal-1.0%	*p*-Value
Laying performance (%)	60.56 ± 6.780	51.95 ± 11.349	59.86 ± 7.197	na
ADFI (g/day)	104.17 ± 12.867	101.24 ± 11.791	105.17 ± 10.161	na
Egg mass (g)	38.25 ± 4.418	32.32 ± 6.657	38.45 ± 3.888	na
FCR	2.77 ± 0.277	3.25 ± 0.797	2.78 ± 0.368	na
Body weight start	1649.17 ± 166.24	1631.82 ± 159.65	1611.59 ± 167.90	0.424
Body weight 16th week	1766.78 ± 197.61	1725.77 ± 199.93	1755.59 ± 170.19	0.506

Con: hens fed the control diet, Sal-0.5%: hens were fed the control diet supplemented with 0.5% of *Salvia officinalis* L., Sal-1.0% hens were fed the control diet supplemented with 1.0% of *Salvia officinalis* L., and na: not applicable.

**Table 5:** Effects of treatments on the performance parameters of laying hens during the 2nd experimental period (*n* = 2 per treatment for the laying performance (%), ADFI, egg mass, and FCR; *n* = 66 per treatment for the body weight measurements).

Parameter	Treatments			
	Con	Sal-0.5%	Sal-1.0%	*p*-Value
Laying performance (%)	57.72 ± 4.366	62.89 ± 4.476	55.49 ± 5.441	na
ADFI (g/day)	111.62 ± 10.622	117.78 ± 7.294	110.46 ± 5.805	na
Egg mass (g)	37.75 ± 3.464	41.44 ± 3.691	36.51 ± 3.453	na
FCR	2.98 ± 0.364	2.86 ± 0.251	3.06 ± 0.402	na
Body weight start	1549.85 ± 191.46	1519.5 ± 169.11	1490.30 ± 138.80	0.128
Body weight 16th week	1832.69 ± 188.90	1840.00 ± 244.49	1789.83 ± 159.90	0.358

Con: hens fed the control diet, Sal-0.5%: hens were fed the control diet supplemented with 0.5% of *Salvia officinalis* L., Sal-1.0%: hens were fed the control diet supplemented with 1.0% of *Salvia officinalis* L., and na: not applicable.

**Table 6:** Effects of treatments on the performance parameters of laying hens for both the 1st and 2nd experimental periods (*n* = 4 per treatment).

Parameter	Treatments			
	Con	Sal-0.5%	Sal-1.0%	*p*-Value
Laying performance (%)	58.67 ± 6.15	57.36 ± 10.08	58.25 ± 6.43	0.620
ADFI (g/day)	108.59 ± 11.59	109.21 ± 12.70	108.17 ± 7.36	0.928
Egg mass (g)	35.85 ± 6.37 ^a^	39.60 ± 4.16 ^b^	36.96 ± 3.58 ^ab^	0.008
FCR	3.14 ± 0.80 ^a^	2.77 ± 0.33 ^b^	2.95 ± 0.38 ^ab^	0.031

Con: hens fed the control diet, Sal-0.5%: hens were fed the control diet supplemented with 0.5% of *Salvia officinalis* L., and Sal-1.0% hens were fed the control diet supplemented with 1.0% of *Salvia officinalis* L. ^a,b^ Values sharing different superscripts differ between them significantly at *p* < 0.05.

**Table 7:** Effects of the treatments on the egg quality parameters during year 1 (*n* = 96 per treatment).

Parameter	Treatments			
	Con	Sal-0.5%	Sal-1.0%	*p*-Value
Egg weight (g)	63.52 ± 5.818 ^x^	63.99 ± 6.202 ^xy^	64.82 ± 6.431 ^y^	0.087
Yolk weight (g)	15.20 ± 1.660 ^a^	15.30 ± 1.675 ^ab^	15.50 ± 1.712 ^b^	0.043
Shell weight (g)	5.99 ± 0.724 ^x^	5.97 ± 0.682 ^x^	6.11 ± 0.725 ^y^	0.060
Albumen weight (g)	42.33 ± 4.671	42.72 ± 5.381	43.15 ± 5.152	0.268
Shell thickness (mm)	0.44 ± 0.039	0.43 ± 0.038	0.44 ± 0.036	0.143
Egg length (mm)	58.06 ± 2.552	58.38 ± 2.443	58.52 ± 2.381	0.138
Egg width (mm)	43.93 ± 1.421 ^x^	43.96 ± 1.488 ^x^	44.24 ± 1.550 ^y^	0.067
Shape index	0.76 ± 0.028	0.75 ± 0.027	0.76 ± 0.027	0.363
Shell color	29.07 ± 4.927	29.41 ± 4.348	29.46 ± 4.964	0.653
Yolk weight	6.94 ± 0.988	6.99 ± 0.827	7.04 ± 0.792	0.546
Haugh units	90.45 ± 9.077	90.62 ± 10.140	89.28 ± 10.368	0.327
Albumen pH	8.62 ± 0.274 ^a^	8.59 ± 0.291 ^a^	8.68 ± 0.214 ^b^	0.001
Yolk pH	6.13 ± 0.158 ^a^	6.09 ± 0.101 ^b^	6.10 ± 0.068 ^b^	0.002
Yolk color	6.94 ± 0.988	7.00 ± 0.889	7.04 ± 0.792	0.466
Specific gravity	1.08 ± 0.007	1.08 ± 0.006	1.08 ± 0.006	0.655

Con: hens fed the control-standard diet, Sal-0.5%: hens were fed the control diet supplemented with 0.5% of *Salvia officinalis* L., and Sal-1.0% hens were fed the control diet supplemented with 1.0% of *Salvia officinalis* L. ^a,b^ Values sharing different superscripts differ between them significantly at *p* < 0.05. ^x,y^ Values sharing different superscripts tend to differ between them at 1 > *p* > 0.05.

**Table 8:** Effects of treatments on the egg quality parameters during year 2 (*n* = 96 per treatment).

Parameter	Treatments			
	Con	Sal-0.5%	Sal-1.0%	*p*-Value
Egg weight (g)	65.37 ± 5.497	65.87 ± 6.411	65.84 ± 5.037	0.597
Yolk weight (g)	15.42 ± 1.226	15.58 ± 1.819	15.61 ± 1.468	0.362
Shell weight (g)	6.13 ± 0.649	6.16 ± 0.756	6.18 ± 0.594	0.695
Albumen weight (g)	43.82 ± 4.612	44.13 ± 5.089	44.04 ± 4.179	0.787
Shell thickness (mm)	0.44 ± 0.035	0.43 ± 0.034	0.44 ± 0.0316	0.428
Egg length (mm)	58.17 ± 1.958	58.42 ± 2.408	58.26 ± 2.087	0.493
Egg width (mm)	44.62 ± 1.409	44.70 ± 1.543	44.77 ± 1.218	0.586
Shape index	0.77 ± 0.025	0.77 ± 0.026	0.77 ± 0.025	0.471
Shell color	25.27 ± 5.384	25.80 ± 4.790	25.23 ± 3.987	0.349
Yolk weight	7.66 ± 0.979	7.77 ± 0.915	7.82 ± 0.852	0.231
Haugh units	91.77 ± 9.992	92.17 ± 8.034	90.41 ± 10.431	0.254
Albumen pH	8.40 ± 0.293 ^a^	8.48 ± 0.232 ^b^	8.52 ± 0.220 ^c^	<0.001
Yolk pH	6.01 ± 0.127 ^a^	6.02 ± 0.109 ^a^	6.06 ± 0.208 ^b^	0.001
Yolk color	7.66 ± 0.979	7.77 ± 0.915	7.82 ± 0.852	0.212
Specific gravity	1.09 ± 0.006	1.09 ± 0.006	1.09 ± 0.007	0.711

Con: hens fed the control-standard diet, Sal-0.5%: hens were fed the control diet supplemented with 0.5% of *Salvia officinalis* L., and Sal-1.0% hens were fed the control diet supplemented with 1.0% of *Salvia officinalis* L. ^a–c^: Values sharing different superscripts differ between them significantly at *p* < 0.05.

**Table 9:** Effects of the treatments on the oxidative stability of egg yolk during year 1 (*n* = 12 per treatment).

Time		Treatments		
	Con	Sal-0.5%	Sal-1.0%	*p*-Value
Τ = 0 min	43.59 ± 7.749	56.23 ± 12.571	41.62 ± 6.538	0.107
T = 50 min	859.71 ± 424.689	951.88 ± 336.77	688.70 ± 104.288	0.521
T = 100 min	772.75 ± 247.486	780.29 ± 103.399	756.52 ± 295.707	0.989
T = 150 min	721.16 ± 272.843	628.99 ± 102.299	561.16 ± 132.086	0.497

Con: hens fed the control-standard diet, Sal-0.5%: hens were fed the control diet supplemented with 0.5% of *Salvia officinalis* L., and Sal-1.0% hens were fed the control diet supplemented with 1.0% of *Salvia officinalis* L.

**Table 10:** Effects of the treatments on the oxidative stability of egg yolk during year 2 (*n* = 12 per treatment).

Time		Treatments		
	Con	Sal-0.5%	Sal-1.0%	*p*-Value
Τ = 0 min	45.78 ± 18.961	55.65 ± 21.167	62.61 ± 30.821	0.630
T = 50 min	582.03 ± 53.818 ^a^	533.33 ± 59.482 ^ab^	448.67 ± 66.819 ^b^	0.034
T = 100 min	585.51 ± 38.917 ^a^	553.62 ± 113.305 ^a^	430.15 ± 48.732 ^b^	0.038
T = 150 min	731.01 ± 168.776 ^a^	513.04 ± 101.913 ^b^	413.33 ± 33.73 ^b^	0.010

Con: hens fed the control-standard diet, Sal-0.5%: hens were fed the control diet supplemented with 0.5% of *Salvia officinalis* L., and Sal-1.0% hens were fed the control diet supplemented with 1.0% of *Salvia officinalis* L. ^a,b^ Values sharing different superscripts differ between them significantly at *p* < 0.05.

**Table 11:** Effects of the treatments on the total number of *Enterobacteriaceae* of eggshells collected during year 1 (*n* = 12 per treatment).

Parameter	Treatments		
	Con	Sal-0.5%	Sal-1.0%
*Enterobacteriaceae* (N/g)	235.0 ± 147.16	179.0 ± 154.95	108.8 ± 130.71
*p*-Value	Con vs. Sal-0.5%	Con vs. Sal-1.0%	Sal-0.5% vs. Sal-1.0%
	0.282	0.032	0.342

Con: hens fed the control-standard diet, Sal-0.5%: hens were fed the control diet supplemented with 0.5% of *Salvia officinalis* L., and Sal-1.0% hens were fed the control diet supplemented with 1.0% of *Salvia officinalis* L.

**Table 12:** Effects of the treatments on the total number of Enterobacteriaceae of the eggshells collected during year 2 (*n* = 12 per treatment).

Parameter	Treatments		
	Con	Sal-0.5%	Sal-1.0%
*Enterobacteriaceae* (N/g)	233.3 ± 149.74	231.0 ± 184.89	123.3 ± 87.00
*p*-Value	Con vs. Sal-0.5%	Con vs. Sal-1.0%	Sal-0.5% vs. Sal-1.0%
	0.863	0.041	0.087

Con: hens fed the control-standard diet, Sal-0.5%: hens were fed the control diet supplemented with 0.5% of *Salvia officinalis* L., and Sal-1.0% hens were fed the control diet supplemented with 1.0% of *Salvia officinalis* L.

## Data Availability

Data are available by the corresponding author upon request.
